# Robust Vision-Based Control of a Rotorcraft UAV for Uncooperative Target Tracking

**DOI:** 10.3390/s20123474

**Published:** 2020-06-19

**Authors:** Shijie Zhang, Xiangtian Zhao, Botian Zhou

**Affiliations:** Research Center of Satellite Technology, Harbin Institute of Technology, Harbin 150080, China; zhaoxiangtian@hit.edu.cn (X.Z.); btzhou.hit@gmail.com (B.Z.)

**Keywords:** UAV, vision-based control, virtual image plane, uncooperative target tracking, gain-switching control

## Abstract

This paper investigates the problem of using an unmanned aerial vehicle (UAV) to track and hover above an uncooperative target, such as an unvisited area or an object that is newly discovered. A vision-based strategy integrating the metrology and the control is employed to achieve target tracking and hovering observation. First, by introducing a virtual camera frame, the reprojected image features can change independently of the rotational motion of the vehicle. The image centroid and an optimal observation area on the virtual image plane are exploited to regulate the relative horizontal and vertical distance. Then, the optic flow and gyro measurements are utilized to estimate the relative UAV-to-target velocity. Further, a gain-switching proportional-derivative (PD) control scheme is proposed to compensate for the external interference and model uncertainties. The closed-loop system is proven to be exponentially stable, based on the Lyapunov method. Finally, simulation results are presented to demonstrate the effectiveness of the proposed vision-based strategy in both hovering and tracking scenarios.

## 1. Introduction

Unmanned aerial vehicles (UAVs) have received growing interest, due to their advantages of vertical take off and landing, rapid maneuverability, and low cost. With improvements in sensing devices, batteries, materials, and other technologies, UAVs have sufficient payload and flight endurance, supporting many applications such as transportation, real-time monitoring, search and rescue, and security and surveillance [1,2,3,4]. There have been a variety of studies related to missions using autonomous hovering and tracking technologies [5,6,7]. In [5], a finite-time controller was proposed to drive a quadrotor hovering above a target with a limited duration. In [6], a novel fuzzy PID-type iterative learning control was developed for trajectory tracking of a quadrotor under the effects of external disturbances and uncertain factors of the system. The problem of energy-efficient path planning and simultaneously anticipating disturbances has been addressed in [7]. However, the available studies have mainly focused on hovering above or tracking a target with a known trajectory and definite position and velocity information. Tracking passive, uncooperative, or even unknown targets, such as vehicles in traffic accidents and fire areas in groves or forests, is still a challenging problem for UAV control. These cases usually occur suddenly and detailed geometric information (e.g., dimensions and size) of the target is not available. On the other hand, rough information (e.g., shape and structure) or a simplified model of the target can be stored onboard, which can help the vehicle to identify and lock on to the target.

A large amount of state of the art sensors has been equipped on the airborne platform, and multi-sensor fusion is a perspective trend in UAV navigation and control [8]. Inertial measurement units (IMUs) and Global Positioning Systems (GPS) are commonly mounted on UAVs to provide position, orientation, and velocity information [9]. However, GPS cannot determine the relative position between the UAV and a target, unless the target’s position information is accurately known. Moreover, the accuracy of GPS is unreliable and ineffective in the presence of obstacles or external disturbances, such as urban canyon effects or electromagnetic interference [10,11]. On the other hand, a large number of UAVs, especially small-scale quadrotors, are equipped with cameras which can provide adequate visual information and act as an alternative option to GPS. The notion of introducing visual information into the control system is referred to as visual servoing [12]. According to different feedback information, visual servoing can be classified into two approaches: position-based visual servoing (PBVS) and image-based visual servoing (IBVS).

PBVS methods rely on relative pose estimation techniques, including the PnP algorithm, epipolar geometry, ICP algorithm, or the fusion of vision and other sensors. In [13], a monocular semi-direct visual odometry algorithm was applied to obtain position information and an adaptive sliding mode controller based on the backstepping technique was presented for quadrotor tracking control. In [14], visual feedback was exploited to estimate the position and attitude of a UAV based on the triangulation method, and a classical PID control was designed for indoor UAV flight. In [15], a vision-based control scheme was proposed for a quadrotor, in order to track a moving target. To compensate for the image dynamic uncertainties, a neural network controller based on a radial basis function was introduced into the closed-loop system. However, the methods mentioned above require a priori knowledge of the target’s geometric model, which does not apply to a newly discovered uncooperative target. Further, 3D reconstruction based on generic features (instead of specific artificial markers) is usually a challenging task, and the sophisticated image processing involved requires long computation times, which may result in delays in the actuation. The IBVS approach, on the other hand, allows for designing a control law directly in the 2D image space and omits the relative pose estimation process. Thus, the geometric information of the target is not required in this approach. The applications of IBVS for UAV control are largely found in recent studies, in which image features have been used as the position cue. To decouple the rotational motion of the vehicle from the change in the image features, several methods have been adopted in IBVS control of a UAV, including spherical projection [16,17], homography-based methods [18,19], virtual spring methods [20,21], and virtual camera methods [22,23]. It is worth noting that the reference image (or image trajectory) is usually designated ahead of the control process, which is typically referred to as the “teach-by-showing” framework. However, this process may not be practical in some specific missions, such as when a UAV is deployed to track a newly discovered target [24].

When tracking an uncooperative target without an accurate geometric model, one effective solution is Simultaneous Localization and Mapping (SLAM). SLAM is a real-time process by which a vehicle computes its localization and simultaneously builds a map of an unknown environment [25]. SLAM techniques are often employed to provide the pose estimation for the PBVS control of the UAV. In [26], a novel estimator based on an Extended Kalman Filter was proposed for multi-sensor fusion, including AHRS, GPS, and a monocular camera. Moreover, a visual SLAM-based scheme has been adopted to provide an accurate estimation of the translational position and velocity, as described in [27]. In [28], a strategy based on visual-inertial SLAM was employed for collision avoidance in a GPS-denied environment. In general, visual SLAM requires long computation times and large amounts of onboard resources, thus typically struggling to provide the rapid pose estimation needed by the UAV guidance system.

This paper presents a simple vision-based strategy to track an uncooperative target which is newly discovered by the UAV. The proposed strategy demonstrates a strict interaction between vision-based metrology and the resulting control. It is assumed that the target’s image is acquired and identified by matching the visual data extracted from the monocular camera with similar information stored on board. Then, a set of image features are well-defined by the image processing unit for the following process. Image centroid is employed for the metrology in the horizontal direction, in spite of the fact that the geometric model is unknown to the UAV. To eliminate the coupling effect of the UAV rotational motion and the change in the image features, we introduce a virtual camera frame. After reprojecting the image features onto the virtual image plane, we can directly estimate the relative horizontal distance between the UAV and the target, based on the perspective projection model. At the same time, the flying height of the UAV can be adjusted by specifying an optimal observation area on the virtual image plane. Motivated by the work in [29], the relative velocity is estimated by a Partial Velocity Evaluation method, which is reliable and computationally effective. Robust methods for the optic flow measurement have been reported in [30,31]. Considering the deviation of the measured mass and inertia matrix from the true values, as well as the disturbances resulting from the wind and other external factors, traditional PID algorithms may not be competent in the UAV controller design, as described in [32,33]. To compensate for the model and disturbance uncertainties and improve robust performance of the system, a gain-switching proportional-derivative (PD) control scheme is proposed in this paper. In this control strategy, the stability and robustness properties of the UAV nonlinear system are guaranteed based on the Lyapunov stability theory. Unlike the sliding mode control, which can be found in [34,35], the transient behavior of the system can be characterized analytically.

The remaining parts of this paper are organized as follows. The problem formulation is given in Section 2, the visual measurement for uncooperative target tracking is discussed thoroughly in Section 3, the gain switching PD controllers are designed in Section 4, the corresponding simulation results are depicted in Section 5 and, finally, the paper is concluded in Section 6.

## 2. Problem Formulation

The problem addressed in this paper corresponds to UAV tracking and hovering scenarios in which the target is newly discovered. The target, which is referred to as “uncooperative”, lacks accurate dimension/size information and real-time communication with the vehicle. It can be identified by the rough information of generic features stored onboard, such as shape and structure. The UAV studied here is a quadrotor equipped with multiple sensors, including an IMU, an ultrasonic sensor, and a monocular camera. A vision-based strategy integrating the metrology and the resulting control is employed to achieve target tracking and hovering observation. The quadrotor is a typical underactuated mechanism with more degrees of freedom than actuations. To analyze the tracking maneuvers, we first define several reference coordinate frames and present the equations of the motion of the quadrotor, then give an overall control framework of tracking and hovering observation.

### 2.1. Quadrotor Model

**Figure 1:**
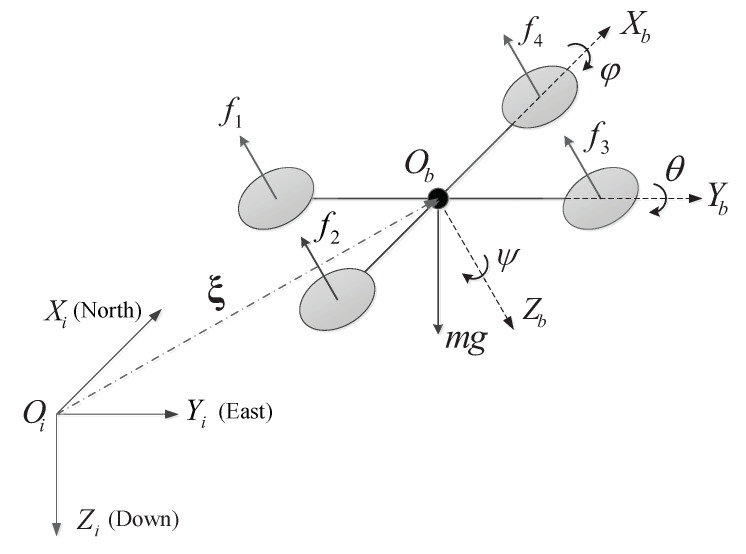
Quadrotor model and frame definitions.

The quadrotor considered in this paper consists of a rigid cross-frame equipped with four rotors, as shown in Figure 1. The two rotors on the diagonal rotate in the same direction, while the adjacent rotors rotate in opposite directions. Six degrees of freedom of the quadrotor’s position and attitude can be achieved by adjusting the rotation speed of the four motors. Two coordinate frames are introduced to describe the equations of motion of the quadrotor. An inertial frame I is fixed to some point $O_{i}$ on the earth, with a basis $\left\{X_{i},Y_{i},Z_{i}\right\}$, whose elements are oriented north, east, and down, respectively. A body-fixed frame B is assumed to be attached to the center of mass $O_{b}$ of the quadrotor. The unit vectors of the body-fixed frame are represented by $\left\{X_{b},Y_{b},Z_{b}\right\}$,which are oriented forward, right, and down, respectively.

Consider a quadrotor with mass *m* and inertial matrix $J\in R^{3\times 3}$. The translational dynamics in the inertial frame and the rotational dynamics in the body frame are given as follows.

For the translational dynamics,

(1) $$\left\{\begin{array}{c} \dot{\xi }=v\\ m\dot{v}=-u_{1}RE_{3}+F_{g}+F_{d} \end{array}\right.,$$

and for the rotational dynamics,

(2) $$\left\{\begin{array}{c} \dot{\Phi }=W\left(\Phi \right)\cdot \omega \\ J\dot{\omega }=-\omega \times J\omega +\tau +\tau_{d} \end{array}\right.,$$

where $\xi ={\left[x,y,z\right]}^{T}$ and $v={\left[v_{x},v_{y},v_{z}\right]}^{T}$ are position and linear velocities of the quadrotor, respectively, expressed in the inertial frame; $E_{3}={\left[0,0,1\right]}^{T}$ is the unit vector in the body frame; the attitude of the vehicle, Φ, is given by three Euler angles φ, θ, and ψ denoting the roll, pitch, and yaw, respectively; and $\omega ={\left[\omega_{x},\omega_{y},\omega_{z}\right]}^{T}$ is the quadrotor’s angular velocity expressed in the body frame. The corresponding rotation matrix from the body frame to the inertial frame is denoted by R, and the matrix associated with the Euler angles and the angular velocity can be written as

(3) $$W\left(\Phi \right)=\left[\begin{array}{ccc} 1 & \sin\varphi \tan\theta & \cos\varphi \tan\theta \\ 0 & \cos\varphi & -\sin\varphi \\ 0 & \sin\varphi /\cos\theta & \cos\varphi /\cos\theta \end{array}\right].$$

**Assumption** **1.** *If the quadrotor does not perform maneuvers that are too aggressive, the roll and pitch angles will both be very small (< $15^{\circ }$). Then, the matrix W(Φ) can be replaced with a unit matrix, and $\dot{\varphi }$, $\dot{\theta }$, $\dot{\psi }$, $\ddot{\varphi }$, $\ddot{\theta }$, $\ddot{\psi }$ can be regarded as approximately equal to $\omega_{x}$, $\omega_{y}$, $\omega_{z}$, ${\dot{\omega }}_{x}$, ${\dot{\omega }}_{y}$, ${\dot{\omega }}_{z}$, respectively.*

According to the working principle of the quadrotor (see Figure 1), by adjusting the rotational speed of each group of rotors, the quadrotor can generate a thrust force $u_{1}$ and torque vectors $\tau ={\left[u_{2},u_{3},u_{4}\right]}^{T}$, which can be described as

(4) $$\left\{\begin{array}{c} u_{1}=b\left(\omega_{1}^{2}+\omega_{2}^{2}+\omega_{3}^{2}+\omega_{4}^{2}\right)\\ u_{2}=bl\left(\omega_{4}^{2}-\omega_{2}^{2}\right)\\ u_{3}=bl\left(\omega_{3}^{2}-\omega_{1}^{2}\right)\\ u_{4}=d\left(\omega_{2}^{2}+\omega_{4}^{2}-\omega_{1}^{2}+\omega_{3}^{2}\right) \end{array}\right.,$$

where $\omega_{1}$, $\omega_{2}$, $\omega_{3}$, and $\omega_{4}$ are the speeds of four motors, respectively; *b* and *d* represent lift and drag coefficients, respectively; and *l* is the distance from the center of each rotor to the center of mass of the quadrotor.

The gravity vector of the quadrotor is denoted by $F_{g}$. As the quadrotor may be disturbed by the wind and other external factors, some unstructured forces and moments of the translational and rotational dynamics, which are described as $F_{d}$ and $\tau_{d}$, are introduced into the system.

**Assumption** **2.** *The external disturbances $F_{d}$ and $\tau_{d}$ are assumed to be bounded; that is, $∥F_{d}∥\leq {\left(F_{d}\right)}_{\max}$ and $∥\tau_{d}∥\leq {\left(\tau_{d}\right)}_{\max}$, where ${\left(F_{d}\right)}_{\max}$ and ${\left(\tau_{d}\right)}_{\max}$ are positive constants and $∥\cdot ∥$ denotes the standard Euclidean vector norm and induced matrix norm.*

**Assumption** **3.** *The mass is $m=\bar{m}+\Delta m$, where $\bar{m}$ and Δm are the nominal and uncertain parts of the mass, respectively. The inertia matrix is $J=\bar{J}+\Delta J$, where $\bar{J}$ and ΔJ are the nominal and uncertain parts of the inertia matrix, respectively. Δm and ΔJ satisfy the inequalities $∥\Delta m∥\leq c_{m}$ and $∥\Delta J∥\leq c_{J}$, where $c_{m}$ and $c_{J}$ are positive constants.*

### 2.2. Control Framework of Tracking and Hovering Observation

**Figure 2:**
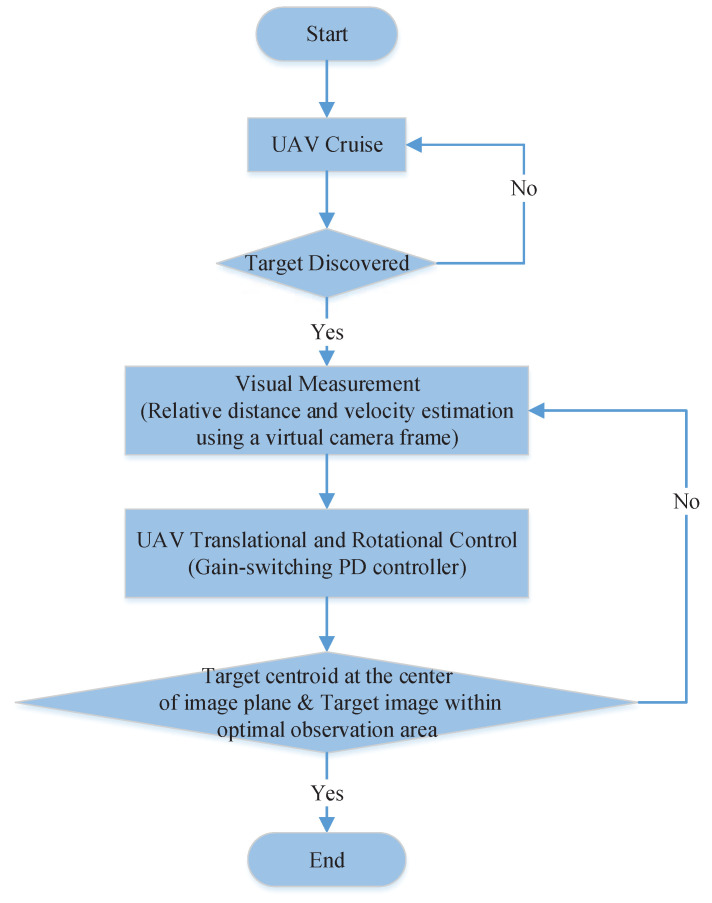
Flowchart of the vision-based unmanned aerial vehicle (UAV) tracking strategy.

A flowchart of the proposed vision-based UAV tracking strategy is presented as follows (see Figure 2). During the cruise of the UAV, when a sensitive target is captured and identified by the onboard camera, the tracking and hovering observation process starts. First, a set of feature points on the target are extracted. By using the center point of the feature points and their pixel velocities, we can determine the relative distance and velocity, respectively, between the UAV and target. Then, the relative distance and velocity are input into the controller as error states. Based on the gain-switching PD control, the target centroid is expected to coincide with the center of the image plane, and the target image is required to be within the optimal observation area.

## 3. Visual Measurement Using a Virtual Camera Frame

When a sensitive target is first detected and determined by the camera during the quadrotor flight, its projection on the image plane can be described by a set of parameters, including slope, curvature, area, image centroid, and some parameters related to the shape of the target [36]. The selected target for tracking is assumed to be horizontal to the *X*${}_{i}$–*Y*${}_{i}$ plane of the inertial frame, and its projection onto the image plane is a compact area with large values of shape parameters, such as sphericity or rectangularity.

### 3.1. Relative Distance Estimation in Horizontal Direction

As the target is uncooperative and cannot send any position information to the quadrotor, the relative distance measurement relies entirely on the vision-based method. However, the target does not have specific markers and its detailed geometry is unknown to the quadrotor, such that it is challenging to use PnP solvers or Template Matching approaches for relative pose acquisition. In this work, we utilize an effective and simple method based on the image error to estimate the relative horizontal distance between the quadrotor and the target. On the other hand, the quadrotor is an underactuated system with only four independently controllable degrees of freedom. Rolling and translational motions along the *Y*${}_{b}$ axis are coupled, as are pitching and translational motions along the *X*${}_{b}$ axis, which means that the vehicle will inevitably tilt when maneuvering horizontally. Thus, the image features of the target will change with not only the translational, but also the rotational, motion of the quadrotor, which makes it more complicated to estimate the relative distance and velocity.

**Figure 3:**
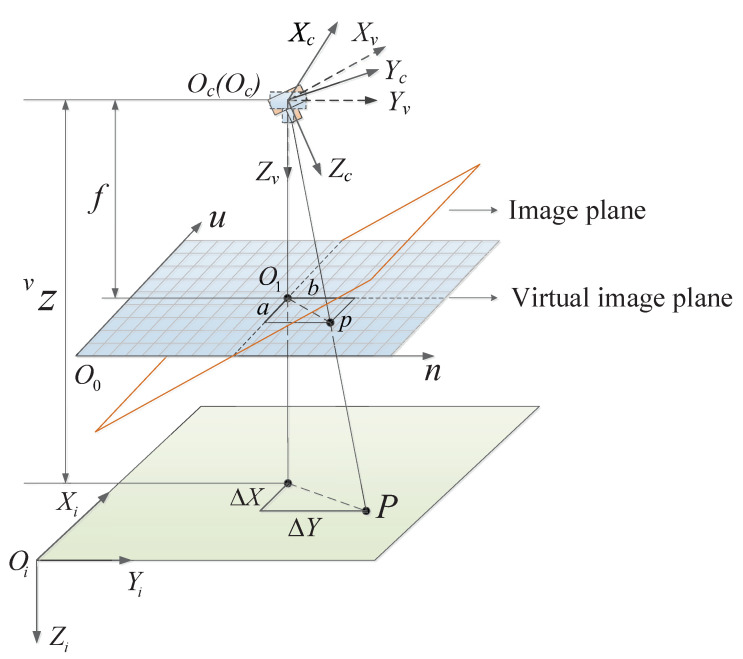
Camera frame C and virtual camera frame V with their corresponding image planes. The perspective projection of point *P* on the virtual image plane.

Without loss of generality, the camera frame considered in this paper, $C=\{X_{c},Y_{c},Z_{c}\}$, is assumed to coincide with the body frame B. To solve the problem mentioned above, we introduce a virtual camera coordinate frame V, which has the same origin as the frame B(C). The corresponding virtual image plane *X*${}_{v}$–*Y*${}_{v}$ is always parallel to the *X*${}_{i}$–*Y*${}_{i}$ plane and with the same yaw angle as the frame C; that is, its roll and pitch angles remain zero (see Figure 3).

The coordinates of a point *P* in the inertial frame and camera frame are denoted by ${}^{i}P={{[}^{i}P_{x}{,}^{i}P_{y}{,}^{i}P_{z}]}^{T}$ and ${}^{c}P={{[}^{c}P_{x}{,}^{c}P_{y}{,}^{c}P_{z}]}^{T}$, respectively. They have the following geometric relationship,

(5) $${}^{c}P=R^{T}\left({}^{i}P-{{}^{i}O}_{c}\right),$$

where ${{}^{i}O}_{c}$ denotes the coordinates of the origin $O_{c}$ in the inertial frame.

The pixel coordinates of the point *P* on the image plane are given by perspective projection equations [37]:

(6) $$\left\{\begin{array}{c} u=f\frac{{}^{c}P_{x}}{{}^{c}P_{z}}+u_{0}\\ n=f\frac{{}^{c}P_{y}}{{}^{c}P_{z}}+n_{0} \end{array}\right.,$$

in which *f* is the focal length of the camera and ${\left[u_{0},n_{0}\right]}^{T}$ is the coordinate of the image plane center.

Now, reproject the image coordinates ${\left[u,n\right]}^{T}$ onto the virtual image plane using a matrix, $R_{\theta \varphi }$, associated with a rotation in the roll angle around *X*${}_{i}$ and in the pitch angle around *Y*${}_{i}$:

(7) $$\left[\begin{array}{c} {}^{v}u\\ {}^{v}n \end{array}\right]=\frac{f}{{\bar{R}}_{\theta \varphi }^{3}s}\left[\begin{array}{c} {\bar{R}}_{\theta \varphi }^{1}p\\ {\bar{R}}_{\theta \varphi }^{2}p \end{array}\right],$$

in which $p={\left[u,n,f\right]}^{T}$ and ${\bar{R}}_{\theta \varphi }^{1}$, ${\bar{R}}_{\theta \varphi }^{2}$, and ${\bar{R}}_{\theta \varphi }^{3}$ are the row vectors of the matrix $R_{\theta \varphi }$.

It is assumed that N(N≥3) non-collinear points are fixed on the selected target. The image centroid of the target is computed as

(8) $${}^{v}u_{g}=\frac{1}{N}\cdot \sum_{k=1}^{N}{}^{v}u_{k},\;\;\;\;\;\;\;\;\;\;\;\;\;\;{}^{v}n_{g}=\frac{1}{N}\cdot \sum_{k=1}^{N}{}^{v}n_{k}.$$

In this paper, we need to drive the quadrotor directly above the target, such that the desired image feature is determined as the center of the virtual image plane, ${{[}^{v}u_{0}{,}^{v}n_{0}]}^{T}$, as shown in Figure 3. The reprojection of the feature points onto the virtual camera frame decouples the pitch and roll motion of the vehicle through the change of coordinates ${{[}^{v}u{,}^{v}n]}^{T}$. Therefore, the relative horizontal distance can be estimated directly, using the deviation of the image centroid of the target from the center of the image plane:

(9) $$\Delta x={}^{v}z\frac{a}{f},\;\;\;\;\;\;\;\;\;\;\;\;\;\;\;\;\;\;\;\Delta y={}^{v}z\frac{b}{f},$$

where $a={}^{v}u_{g}-{}^{v}u_{0}$ and $b={}^{v}n_{g}-{}^{v}n_{0}$ are image errors defined in image space, and ${}^{v}z$ is the vertical distance of the virtual camera frame obtained by the ultrasonic sensor measurement.

### 3.2. Relative Distance Estimation in Vertical Direction

When tracking an uncooperative target, the relative horizontal distance estimated in the above section should converge to and remain at zero. The height control is relatively flexible, depending on different observation requirements. The easiest way which comes to our mind is to keep the quadrotor flying at a constant height; however, this cannot guarantee the target being observed in an optimal area on the image plane. Intuitively, the image size of the target varies with the height of the camera. The quadrotor is required to fly neither too high nor too low, avoiding making the target image invisible or leaving the field of view.

Now, introduce a circle as the optimal observation area on the virtual image plane centered at $O_{1}$ with radius $r_{opt}$. Then, construct a circle that passes through the farthest feature point from the image centroid of the target. The radius of the constructed circle, denoted by *r*, is defined as

(10) $$r=\max\{\sqrt{{{(}^{v}u_{k}-{}^{v}u_{g})}^{2}+{{(}^{v}n_{k}-{}^{v}n_{g})}^{2}}\},k=1,...,N.$$

According to Equation (10), the constructed circle will cover all of the feature points of the selected compact target. Given that the relative horizontal error converges to zero, the vertical distance of the quadrotor can be controlled by adjusting *r* to be equal to or less than $r_{opt}$, which guarantees the target image being kept in the optimal observation area. Therefore, the desired value of radius *r* satisfies the following conditions,

(11) $$r_{d}=\sigma r_{opt},\;\;\;\;\;\;\;\;\sigma \leq 1,$$

where σ is the radius scaling factor and σ=1 indicates that the optimal observation area circumscribes the target image.

From Equations (10) and (11), the desired flying height of the quadrotor can be written as

(12) $$z_{d}={}^{v}z\cdot \frac{r}{r_{d}}={}^{v}z\cdot \frac{r}{\sigma r_{opt}}.$$

Then, we can define the position error in the vertical direction as

(13) $$\Delta z={}^{v}z-z_{d}.$$

To control the translational motion of the quadrotor, we define the full position error as $\Delta \xi ={\left[\Delta x,\Delta y,\Delta z\right]}^{T}$, which is desired to converge to ${\left[0,0,0\right]}^{T}$.

### 3.3. Relative Velocity Estimation

Generally, the control actions require knowledge of not only position error but also velocity error. The quadrotor is equipped with an IMU providing the angular velocity and acceleration of the vehicle. If the target is stationary, the relative velocity can be obtained directly by integrating the measured acceleration information. However, while the precision of the gyro is satisfactory for the needs of the vehicle maneuvers, UAV-equipped accelerometers are usually not accurate enough for evaluating the platform velocity [29]. Velocity error acquirement is more complicated when the uncooperative target is moving. In this paper, we use the optic flow and gyro measurements to estimate the relative quadrotor-to-target velocity, which is recorded by Partial Velocity Evaluation.

Taking the first derivative of Equation (7), we obtain the dynamics of an image feature ${}^{v}p_{k}={\left[{}^{v}u_{k},{}^{v}n_{k}\right]}^{T}$ [22]:

(14) $${}^{v}{\dot{p}}_{k}=L_{vk}\left({}^{v}v-{}^{v}v_{pk}\right)+L_{\psi k}\dot{\psi },$$

in which ${}^{v}v$ and ${}^{v}v_{pk}$ are the velocities of the point *P* and the quadrotor expressed in the virtual camera frame, and the matrices $L_{vk}$ and $L_{\psi k}$ are defined as

$$L_{vk}=\left[\begin{array}{ccc} -\frac{f}{{}^{v}P_{z}} & 0 & \frac{{}^{v}u_{k}}{{}^{v}P_{z}}\\ 0 & -\frac{f}{{}^{v}P_{z}} & \frac{{}^{v}n_{k}}{{}^{v}P_{z}} \end{array}\right],\;L_{\psi k}=\left[\begin{array}{c} {}^{v}n_{k}\\ -{}^{v}u_{k} \end{array}\right].$$

Define a vector of image features, ${}^{v}p={\left[{}^{v}p_{1}^{T},\cdots ,{}^{v}p_{N}^{T}\right]}^{T}\in R^{2N}$, and assume that the velocities of *N* points are approximately equal to the target velocity, ${}^{v}v_{pk}={}^{v}v_{t}$. Then, Equation (14) can be extended as

(15) $${}^{v}\dot{p}=L_{v}\left({}^{v}v-{}^{v}v_{p}\right)+L_{\psi }\dot{\psi },$$

in which $L_{v}={\left[L_{v1}^{T},\cdots ,L_{vN}^{T}\right]}^{T}\in R^{2N\times 3}$ and $L_{\psi }={\left[L_{\psi 1}^{T},\cdots ,L_{\psi N}^{T}\right]}^{T}\in R^{2N}$.

The finite time difference of image features, ${}^{v}\dot{p}$, can be computed by directly measuring the optic flow of the visual features in images. Denoting by Δv the relative quadrotor-to-target velocity expressed in the inertial frame, we have

(16) $$\Delta v=R_{\psi }L_{v}^{+}\left({}^{v}\dot{p}-L_{\psi }\dot{\psi }\right),$$

in which $L_{v}^{+}\in R^{3\times 2N}$ is the Moore–Penrose pseudo-inverse of the matrix $L_{v}$ and $R_{\psi }$ is the rotation matrix corresponding to the yaw angle by the relation $R=R_{\psi }R_{\theta \varphi }$.

The relative translational velocity (expressed in the inertial frame) Δv and the pixel velocities ${}^{v}\dot{p}$ are related by the interaction matrix $L_{vk}$ and $L_{\psi k}$. At least two points on the target are required to determine the three components of vector Δv. Due to the fact that the virtual image plane is introduced, the depth information of each feature point is not necessary, which is an advantage over traditional visual odometry.

## 4. Gain-Switching PD Controller Design

**Figure 4:**
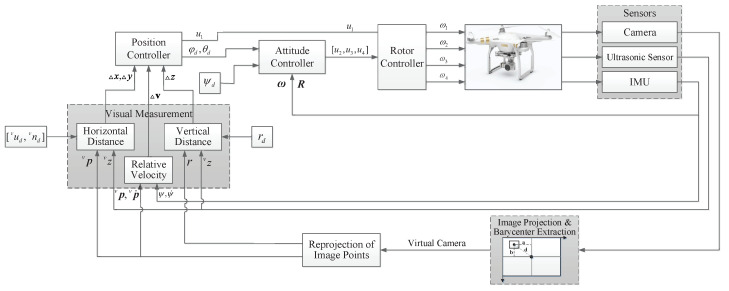
Block diagram of the UAV control system.

The control objective in this work is to drive a quadrotor tracking an uncooperative target at an appropriate height for optimal observation. The quadrotor, as an underactuated system, has six degrees of freedom with only four control inputs. Therefore, the control of a quadrotor is typically divided into an outer position loop and an inner attitude loop. The outer loop provides the reference attitude signal to the inner loop, while the inner loop tracks the orientation reference of the vehicle. A block diagram of the UAV control loop structure is shown in Figure 4. The objective is equivalent to designing a control input for translational motion based on visual feedback, such that the controlled underactuated system with model uncertainties can guarantee Δξ→0 and Δv→0, then designing a control input for rotational motion with the knowledge of reference Euler angles derived from the outer loop.

PD controllers are commonly used in UAV control. Given the uncertainties in the dynamics of the vehicle, a traditional PD controller can produce large steady-state errors or even affect the overall stability of the system. To improve the robustness of the PD controller, we introduce a gain-switching term to deal with the uncertainties by avoiding using large gains.

### 4.1. Control of the Translational Motion

The translational dynamics (1) can be rewritten, in matrix form, as

(17) $$\left[\begin{array}{c} \ddot{x}\\ \ddot{y}\\ \ddot{z} \end{array}\right]=-\frac{u_{1}}{m}\left[\begin{array}{c} \cos\varphi \sin\theta \cos\psi +\sin\varphi \sin\psi \\ \cos\varphi \sin\theta \sin\psi -\sin\varphi \cos\psi \\ \cos\varphi \cos\theta \end{array}\right]\;\;+\left[\begin{array}{c} 0\\ 0\\ g \end{array}\right]+\left[\begin{array}{c} f_{dx}\\ f_{dy}\\ f_{dz} \end{array}\right].$$

The translational motion of the quadrotor is actually accomplished by both the thrust and orientation of the body. Let us define a set of virtual control inputs for the translational motion,

(18) $$\left\{\begin{array}{c} u_{x}=u_{1}\left(\cos\varphi \sin\theta \cos\psi +\sin\varphi \sin\psi \right)\\ u_{y}=u_{1}\left(\cos\varphi \sin\theta \sin\psi -\sin\varphi \cos\psi \right)\\ u_{z}=u_{1}\left(\cos\varphi \cos\theta \right) \end{array}\right..$$

From Equations (17) and (18), we have

(19) $$m\dot{v}=u+F_{g}+F_{d}.$$

The control law for the translational motion is proposed as

(20) $$u=u_{R}+u_{P}+u_{D}+u_{\varepsilon }=-{\bar{F}}_{g}+\left(-K_{P}\bar{m}\Delta \xi \right)+\left(-K_{D}\bar{m}\Delta v\right)+\left(-K_{\varepsilon }\bar{m}n\left(\varepsilon ,s\right)\right),$$

where quantities with overbar symbols indicate that *a priori* estimates are used, which may deviate from the true value; Δξ and Δv are obtained by visual measurement, as detailed in Section 3; $-{\bar{F}}_{g}$ is the model compensation term designed to eliminate nonlinear elements of the dynamics; and $K_{P}$, $K_{D}$, and $K_{\varepsilon }$ represent the proportional, differential, and gain-switching coefficient matrices, respectively. The switching function n(ε,s) can be any piecewise-continuous function with the following properties,

(21) sn(ε,s)=n(ε,s)sn(ε,s)≥1−εεss,s≠0.

In this paper, the switching function is chosen as

(22) n=−srε,r<εε,s<ε−ssss,s≥ε,

where ε is the gain switching threshold and s is the error feedback term given by

(23) $$s=k_{P}\Delta \xi +k_{D}\Delta v.$$

Substituting Equation (20) into (19) yields the error dynamics for the translational motion:

(24) $$m\Delta \dot{v}=-K_{P}m\Delta \xi -K_{D}m\Delta v-K_{\varepsilon }n+h,$$

where the disturbance function, denoted by h, can be written as:

(25) $$h=-K_{P}\delta m\Delta \xi -K_{D}\delta m\Delta v-\delta F_{g}-m\dot{v_{t}}+F_{d},$$

where $\delta m=\bar{m}-m$ and $\delta F_{g}={\bar{F}}_{g}-F_{g}$ denote the deviation between the measured values and the true values, $\dot{v_{t}}$ is the target acceleration, which is assumed to be unknown but has an upper bound.

**Remark** **1.** *The disturbance function h represents all sources of uncertainties in translational error dynamics, including model uncertainty resulting from the inaccurately measured mass of the quadrotor $-K_{P}\delta m\Delta \xi$ and $-K_{D}\delta m\Delta v$, gravitational error $-\delta F_{g}$, unknown target motion $-m\dot{v_{t}}$, and unstructured forces $F_{d}$. Under Assumption 2–3, the disturbance function h is bounded.*

Now, the properties of the gain-switching coefficient will be addressed. The matrix $K_{\varepsilon }$ is symmetric and positive definite, and must satisfy the following conditions,

(26) KεnKεn(m¯+cm)(m¯+cm)≥kεnkε≥hh(m¯+cm)(m¯+cm),

where $k_{\varepsilon }$ is a positive bounding scalar designated to ensure that the term $u_{\varepsilon }$ provides greater acceleration than that resulting from the disturbances in h.

The proportional and differential coefficient matrices $K_{P}$ and $K_{D}$ are selected to satisfy the following constraints with $k_{P}$ and $k_{D}$,

(27) KPm¯ΔξKPm¯Δξ(m¯+cm)(m¯+cm)≥kPΔξKDm¯ΔvKDm¯Δv(m¯+cm)(m¯+cm)≥kDΔv,

in which $k_{P}$ and $k_{D}$ are feedback gains designated as the following functions of the desired rate of convergence, α:

(28) $$\left\{\begin{array}{c} k_{P}=2\alpha^{2}\\ k_{D}=2\alpha \end{array}\right.,\;\;\;\;\;\;\;\;\;\;\;\alpha >0.$$

These equations guarantee that the norm of $K_{P}$ and $K_{D}$ is large enough, such that the commanded force specified by $u_{P}$ and $u_{D}$ delivers—at a minimum—the acceleration specified by the vector $u_{\varepsilon }$.

**Remark** **2.** *Compared with traditional PD control, the proposed PD control consists of not only the proportional and differential terms $u_{P}$ and $u_{D}$, which can eliminate the position and velocity errors, but also a gain-switching term $u_{\varepsilon }$, which acts as a robustifying term based on the switching function n(ε,s). By using Equation *(26)*, appropriate robust control parameters can be selected to restrain the uncertainties h. In addition, the transient behavior of the system can be characterized analytically, based on the desired rate of convergence in feedback gains *(28)*.*

Referring to Equation (24) and defining the system state $e_{t}={\left[\Delta \xi^{T},{\Delta v}^{T}\right]}^{T}$, the tracking dynamics model for translational motion can be written as

(29) $$\left[\begin{array}{c} \Delta \dot{\xi }\\ \Delta \dot{v} \end{array}\right]=\left[\begin{array}{c} \Delta v\\ \left(\frac{1}{m}\right)\cdot \left(-K_{P}m\Delta \xi -K_{D}m\Delta v-K_{\varepsilon }n\left(\varepsilon ,s\right)+h\right) \end{array}\right].$$

**Lemma** **1.** *Consider a system $\dot{x}\left(t\right)=f\left(t,x\left(t\right)\right)$. If there exist a continuously differentiable function V(x) and scalars $c_{1},c_{2}>0$, which satisfy [38]*

$$\begin{array}{c} \left(i\right)\;\;\;\;\;\;\;\;\;\;c_{1}{∥x∥}^{2}\leq V\left(x\right)\leq c_{2}{∥x∥}^{2}\\ \left(\mathrm{ii}\right)\;\;\;\;\;\;\;\;\;\forall x\in \{x|\;\;\;V\left(x\right)>V^{*},V^{*}>0\},\\ \;\;\;\;\;\;\;\;\;\;\;\;\;\;\;\;\;\;\;\;\;\;\;\exists \dot{V}\left(x\right)\leq -2\alpha \left[V\left(x\right)-V^{*}\right], \end{array}$$

*then the system is (globally and uniformly) exponentially convergent to B(q) with rate α, where*

B(q)=Δ{x∈Rn:x≤q}q=Δ(V*V*c1c1)1/2.

**Theorem** **1.** *Consider the closed-loop system for the translational motion in Equation *(29)* with the controller designed by Equation *(20)*. If the corresponding parameters are assigned as in Equations *(26)*–*(28)*, then the state $e_{t}$ will exponentially converge to zero.*

**Proof.**  The Lyapunov function candidate is designated as

(30) $$V\left(e_{t}\right)=\frac{1}{2}e_{t}^{T}Ae_{t},$$

where

(31) $$A=\left[\begin{array}{cc} k_{L}I & k_{P}I\\ k_{P}I & k_{D}I \end{array}\right]$$

and I is a unit matrix. The constant parameter $k_{L}$ is also a function of α:

(32) $$k_{L}=4\alpha^{3}.$$

By using the values $k_{P}$, $k_{D}$, and $k_{L}$ in Equations (28) and (32), the matrix A is positive definite. Thus, $V(e_{t})$ is a positive definite function satisfying

(33) $$\lambda_{\min}{∥e_{t}∥}^{2}\leq V\left(e_{t}\right)\leq \lambda_{\max}{∥e_{t}∥}^{2}.$$

Condition (i) in Lemma 1 is satisfied with $c_{1}=\lambda_{\min}$ and $c_{2}=\lambda_{\max}$, where $\lambda_{\min}$ and $\lambda_{\max}$ are the smallest and largest eigenvalues of A.

Taking the time derivative of $V(e_{t})$ and substituting Equation (29) into it, we have

(34) V˙(et)=kLΔξTΔv+kPΔvTΔv−kP[ΔξTKPΔξ+ΔξTKDΔv]−kD[ΔvTKDΔv+ΔvTKPΔξ]+(11mm)sT(h−Kεn(ε,s)).

Using the parameter constraints in Equations (26) and (27), it follows that

(35) V˙(x)≤−kP2ΔξTΔξ+(kP−kD2)ΔvTΔv+(kL−2kPkD)ΔξTΔv+sT[(11mm)h−kεn(ε,s)].

Substituting Equations (28) and (32) into (35) leads to the following,

(36) $$\dot{V}\left(e_{t}\right)\leq -2\alpha V\left(e_{t}\right)+E,$$

where

(37) E=sT[(11mm)h−kεn(ε,s)].

By using the properties of n in (21), Equation (37) yields the following expression,

(38) $$E\leq ∥s∥h^{*}-k_{\varepsilon }∥n∥∥s∥,$$

where h*=hhmm.

Equation (22) requires that n=ssεε for $∥s∥<\varepsilon$. Moreover, recalling (26), which states that $k_{\varepsilon }\geq h^{*}$, Equation (38) shows, in this case, that

(39) $$E\leq k_{\varepsilon }\cdot \Delta ,$$

where Δ=(ε2−s2)(ε2−w2)εε>0.

When $∥s∥\geq \varepsilon$, n=−ssss, it follows that

(40) E≤0.

Combining Equations (39) and (40), we can obtain a global upper bound

(41) $$\bar{E}\leq k_{\varepsilon }\cdot \Delta .$$

Substituting Equation (41) into the time derivative of $\dot{V}\left(e_{t}\right)$ in Equation (38) leads to

(42) $$\dot{V}\left(e_{t}\right)\leq -2\alpha \left[V\left(e_{t}\right)-V^{*}\right],$$

in which V*=E¯E¯2α2α.

Inequality (42) ensures that the state of the system $e_{t}$ can exponentially converge to a small ball around the origin defined by $V\left(e_{t}\right)<V^{*}$. Thus, the condition (ii) of Lemma 1 is satisfied. □

It is worth noting that the control law given by Equation (20) yields virtual control inputs for translational motion. Using Equation (18), we can compute the command thrust $u_{1}$, as well as the desired roll and pitch angles for the attitude controller:

(43) $$\left\{\begin{array}{c} u_{1}=\sqrt{u_{x}^{2}+u_{y}^{2}+u_{z}^{2}}\\ \varphi_{d}=\arcsin\left(\frac{u_{x}\sin\psi_{d}-u_{y}\cos\psi_{d}}{u_{1}}\right)\\ \theta_{d}=\arctan\left(\frac{u_{x}\cos\psi_{d}+u_{y}\sin\psi_{d}}{u_{z}}\right) \end{array}\right.,$$

where $\psi_{d}$ is the reference value of yaw. As the yaw angle is independent of the outer loop, we can prescribe it as its initial value or another constant.

### 4.2. Control of the Rotational Motion

In the inner attitude loop, a similar gain-switching PD control law is presented:

(44) $$\begin{array}{cc} \tau = & \tau_{R}+\tau_{P}+\tau_{D}+\tau_{\varepsilon }\\ = & \omega \times \bar{J}\omega +\left(-K_{P}^{\prime }\bar{J}\Delta \Phi \right)+\left(-K_{D}^{\prime }\bar{J}\Delta \omega \right)+\left(-K_{\varepsilon }^{\prime }\bar{J}n^{\prime }\left(\varepsilon^{\prime },s^{\prime }\right)\right), \end{array}$$

where $\Delta \Phi =\Phi -\Phi_{d}$ and $\Delta \omega =\omega -\omega_{d}$ denote the quadrotor attitude and the equivalent rate error, respectively, and $\Phi_{d}={\left[\varphi_{d},\theta_{d},\psi_{d}\right]}^{T}$ are the desired Euler angles output from the outer loop. The desired angular velocity $\omega_{d}$ is taken to be zero.

The switching function $n^{\prime }\left(\varepsilon^{\prime },s^{\prime }\right)$ is chosen the same as before, and the error feedback term $s^{\prime }$ is given by

(45) $$s^{\prime }=-k_{P}^{\prime }\Delta \Phi -k_{D}^{\prime }\Delta \omega .$$

Referring to Equation (2) and defining the system states $e_{r}={\left[\Delta \Phi^{T},{\Delta \omega }^{T}\right]}^{T}$, the tracking dynamics model for rotational motion, under Assumption 1, can be written as

(46) $$\left[\begin{array}{c} \Delta \dot{\Phi }\\ \Delta \dot{\omega } \end{array}\right]=\left[\begin{array}{c} \Delta \omega \\ J^{-1}\left(-K_{P}^{\prime }J\Delta \Phi -K_{D}^{\prime }J\Delta \omega -K_{\varepsilon }^{\prime }Jn^{\prime }\left(\varepsilon^{\prime },s^{\prime }\right)+h^{\prime }\right) \end{array}\right],$$

where the disturbance function $h^{\prime }$ is given by

(47) $$h^{\prime }=-K_{P}^{\prime }\delta J\Delta \Phi -K_{D}^{\prime }\delta J\Delta \omega +\left(\omega \times \bar{J}\omega -\omega \times J\omega \right)-J{\dot{\omega }}_{t}+\tau_{d}.$$

**Theorem** **2.** *Consider the closed-loop system for the rotational motion in Equation *(46)* with the controller designed by Equation *(44)*. If the corresponding parameters are assigned by*

(48) KP′J¯ΔΦKP′J¯ΔΦλmax(J)λmax(J)≥kP′ΔΦKD′J¯ΔωKD′J¯Δωλmax(J)λmax(J)≥kD′Δω,

(49) K′εK′ελmax(J)λmax(J)≥kε′n′(ε′,s′)kε′≥h′h′λmin(J)λmin(J),

(50) $$\left\{\begin{array}{c} k_{P}^{\prime }=2{\alpha^{\prime }}^{2}\\ k_{D}^{\prime }=2\alpha^{\prime } \end{array}\right.,$$

*then, the state $e_{r}$ will exponentially converge to zero.*

**Proof.**  The stability analysis of the system for rotational motion is similar to that for translational motion, so it is not described in detail here. □

## 5. Simulation Results

In this section, MATLAB simulations are presented to validate the performance of the proposed vision-based control scheme. We considered two scenarios in this work: hovering above a stationary target and tracking a moving target. In both scenarios, the target to be observed was assumed to be uncooperative and without detailed geometry information. The physical parameters of the simulated quadrotor were m=2.1 kg and J=diag{0.0096,0.0098,0.016} $\mathrm{kg}\cdot m^{2}/\mathrm{rad}$. The nominal part of the quadrotor’s mass and moment of inertia were $\bar{m}=2.1$ kg and $\bar{J}=\mathrm{diag}\left\{0.0081,0.0081,0.0142\right\}$ $\mathrm{kg}\cdot m^{2}/\mathrm{rad}$. The focal length divided by pixel size of the camera was set as 213, and the image resolution was 160×120 pixels with principal point located at [80,60]. Considering that the quadrotor may be affected by wind disturbances in the environment, we applied some sinusoidal (cosinusoidal) forces and torques to the vehicle with the following values,

$$\begin{array}{c} F_{d}=0.5\cdot {\left[\begin{array}{ccc} \sin(2\pi t) & \cos(\pi t) & -\sin(2\pi t) \end{array}\right]}^{T}\\ \tau_{d}=0.01\cdot {\left[\begin{array}{ccc} -\sin(2\pi t) & -\cos(\pi t) & \sin(2\pi t) \end{array}\right]}^{T} \end{array}.$$

### 5.1. Scenario 1: Hovering Observation

In some cases, such as traffic and fire accidents, we usually drive a UAV equipped with optical sensors to approach the scene of the accident and hover over the damaged vehicle or burning object. To provide distinct images and effective air support for the subsequent rescue, it is required to adjust the height of the UAV, keeping the designated target within the optimal area of the camera’s field of view. The proposed vision-based control scheme in this work is applicable to the above missions.

In the simulation, the target was set as a rectangular object. Its visual features included its four vertexes with the following coordinates, [0.2,0.25,0] m, [0.2,0.25,0] m, [−0.2,−0.25,0] m, and [0.2,−0.25,0] m, all expressed in the inertial frame. The quadrotor initial position and attitude were [1,0.8,−4] m and [0,0,0.2] rad, respectively. For the optimal observation of the target, we defined a reference circle of radius 40 pixels on the virtual image plane, and the radius scaling factor σ was designated to be 1. The corresponding desired height was 1.7048 m, which can be computed using Equation (12). The control gains used in the simulation are listed in Table 1.

The transient response of the system mainly depends on the feedback gains $k_{P}$, $k_{D}$ and $k_{P}^{\prime }$, $k_{D}^{\prime }$, which are determined by the constraint Equations (28) and (50). Based on the trial and error method, the desired rates of convergence for translational and rotational motion were selected as $\sqrt{2}/2$ and 2, respectively. Then, the proportional and differential matrices $K_{P}$, $K_{D}$ and $K_{P}^{\prime }$, $K_{D}^{\prime }$ could be determined by using Equations (27) and (48). To guarantee the robustness properties of the system in the presence of wind and modelling errors, the control gains in the gain-switching term $k_{\varepsilon }$, $K_{\varepsilon }$ and $k_{\varepsilon }^{\prime }$, $K_{\varepsilon }^{\prime }$ must be selected such that they are larger than the effect resulting from the unstructured disturbances h, $h^{\prime }$, based on Equations (26) and (49). The gain switching thresholds ε and $\varepsilon^{\prime }$ were tuned repeatedly to restrain the chattering phenomenon of the control efforts.

**Figure 5:**
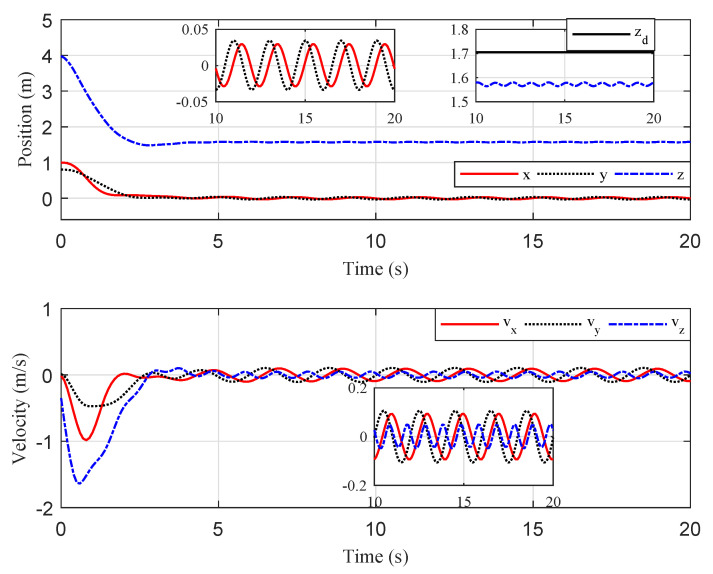
Scenario 1: Time evolution of the quadrotor Cartesian coordinates and velocity.

**Figure 6:**
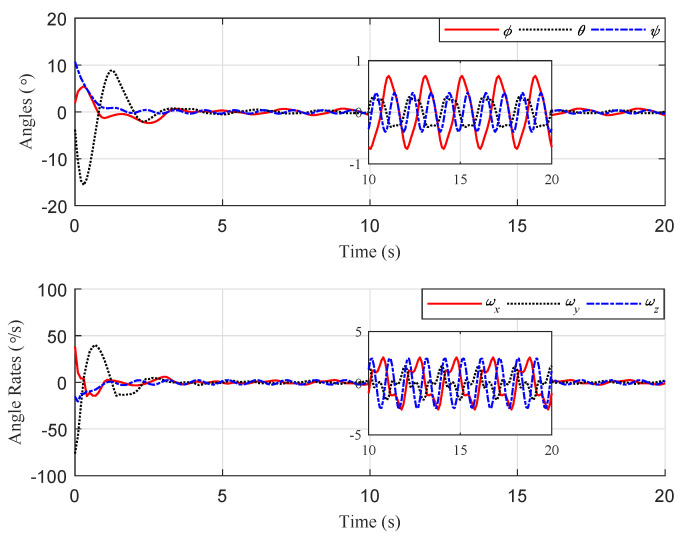
Scenario 1: Time evolution of the quadrotor attitude and angular velocity.

**Figure 7:**
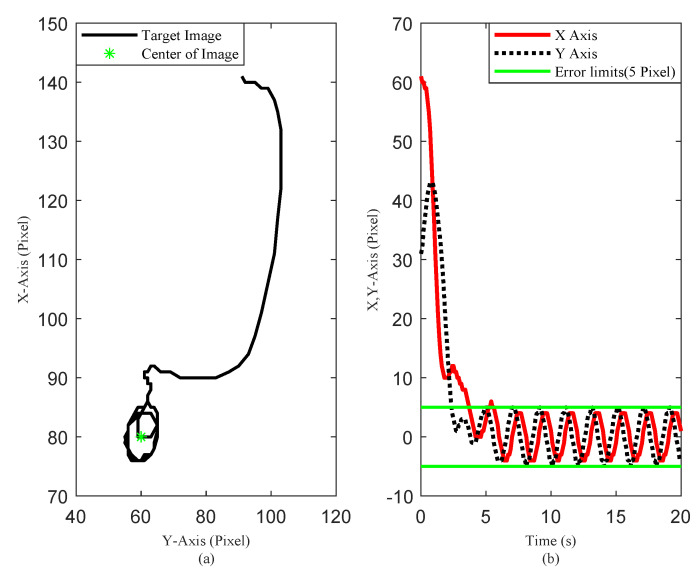
Scenario 1: (**a**) Target centroid trajectory on the image plane. (**b**) Target centroid deviation from the image center.

The simulation results are illustrated in Figure 5, Figure 6 and Figure 7. The translational motion of the quadrotor is shown in Figure 5. The horizontal position errors converged to less than 0.05 m with transient response time enduring at about 5 s. The error in the vertical direction was relatively large, about 0.12 m, mainly resulting from the uncertainty of the quadrotor mass. Figure 6 describes the rotational motion of the quadrotor. The Euler angles were kept within a small range, satisfying Assumption 1. The properties of the target centroid in image space are illustrated in Figure 7. It is shown that the vehicle could rapidly and smoothly approach the target, and the control accuracy of the target centroid on the image plane was kept within 5 pixels. The proposed system, therefore, is satisfactory for continuous hovering observation of the target.

**Figure 8:**
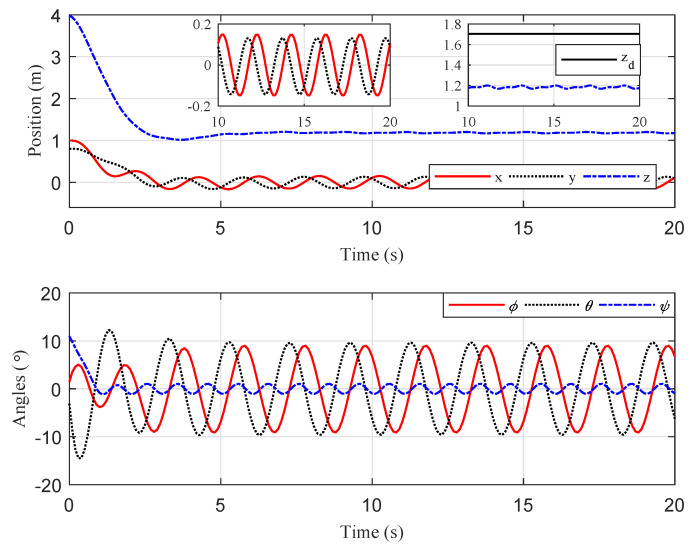
Scenario 1: Time evolution of the quadrotor Cartesian coordinates and attitude (proportional-derivative (PD) controller).

**Figure 9:**
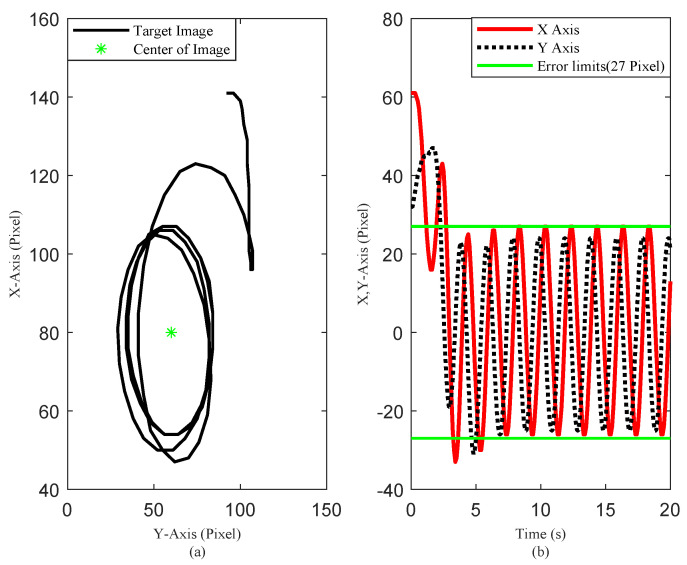
Scenario 1: (**a**) Target centroid trajectory on the image plane; and (**b**) Target centroid deviation from the image center (PD controller).

For comparison, we ran a simulation of traditional PD control using the same initial conditions and gains. The results are reported in Figure 8 and Figure 9. The results indicate that the proposed gain-switching PD controller had better performance than the traditional PD controller in steady-state performance, with lower oscillation, and the proposed method had a smaller error limit of the target centroid in image space. Further, we relocated the quadrotor at two different initial positions to compare the system steady-state behavior between the gain-switching PD controller with the traditional PD controller. The mean, amplitude, and accuracy (3σ) of the position error were illustrated in Table 2. The results showed that the proposed controller was effective in different initial conditions. Compared with the traditional PD controller, the performance of the proposed controller was greatly improved, with the position-control accuracy increasing by a factor of 4–5.

**Figure 10:**
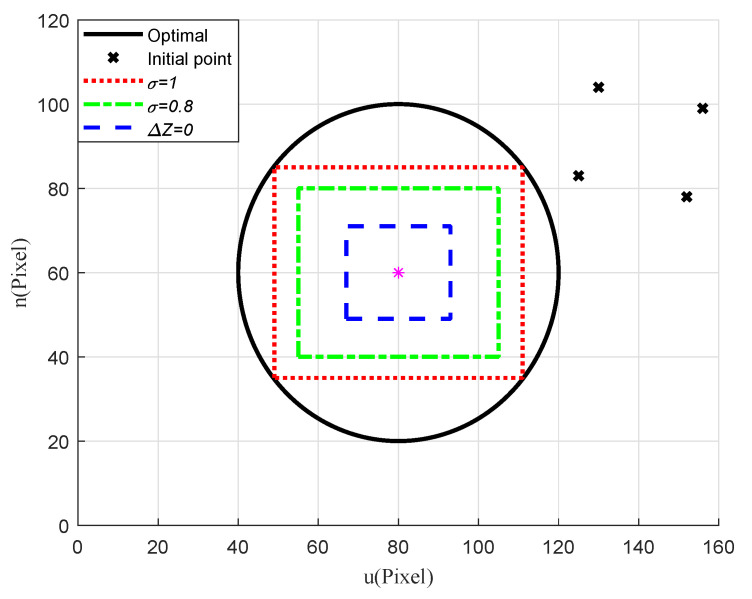
Scenario 1: Imaging effect of the rectangular target under different observation requirements.

**Figure 11:**
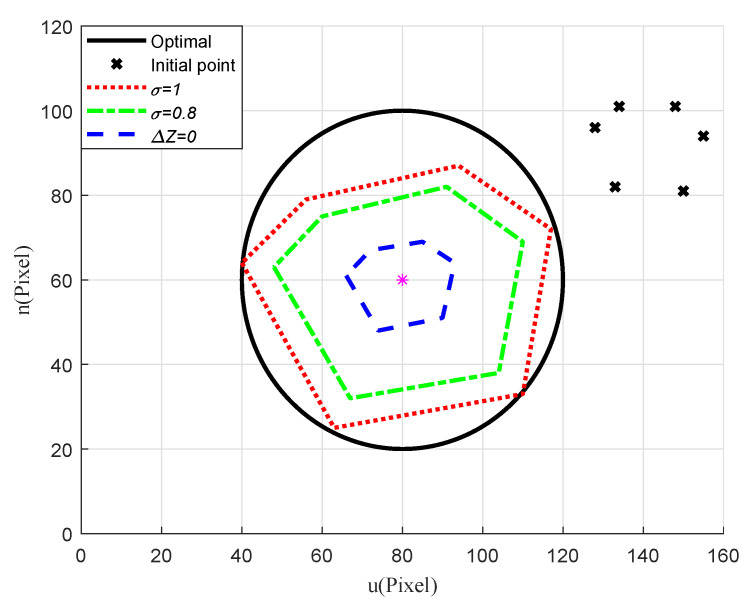
Scenario 1: Imaging effect of the irregularly shaped target under different observation requirements.

To evaluate the imaging effects of the target and the corresponding flying height of the quadrotor under different observation requirements, we ran another simulation ignoring the external disturbance and model uncertainties. In the simulation, three criteria were set: flying at a constant height (i.e., Δz=0), radius scaling factor σ=1, and σ=0.8. As plotted in Figure 10, the results showed that introducing a reference circle on the virtual image plane and adjusting the radius scaling factor can regulate the imaging effect, according to different observation requirements. To illustrate that the proposed strategy is applicable to different kinds of targets, we ran another simulation to hover above an irregularly shaped target. The Cartesian coordinates of the feature points that enclose the target were: [0.25,−0.05,0] m, [0.10,0.20,0] m, [−0.20,0.15,0] m, [−0.25,−0.10,0] m, [−0.10,−0.20,0] m, [0.15,−0.15,0] m. The imaging effect under different observation requirements was shown in Figure 11. Similar to the rectangular target, the proposed control strategy can meet the hovering and observation needs of the target.

### 5.2. Scenario 2: Tracking a Moving Target

We also considered some more challenging cases, such as when the target vehicle is moving or when a fire area changes in real-time. Therefore, not only positioning the UAV over the target is required, but also following its (unknown) trajectory. Compared with the traditional PD control, the robust controller proposed in this paper was more competent in this challenging mission. In this simulation, the target had the same geometric characteristics as in SCENARIO 1 but followed three different trajectories on the flat ground: circular movement, S-type movement, and linear movement. The quadrotor initial position was set to be [1,1.5,−3] m. To evaluate the performance of the proposed controllers in a more realistic environment, we added some noise to the measurement information. White noise with covariances of 0.5 and 10${}^{-4}$ was augmented to the visual data (image features and their pixel velocities) and angular rates, respectively.

**Figure 12:**
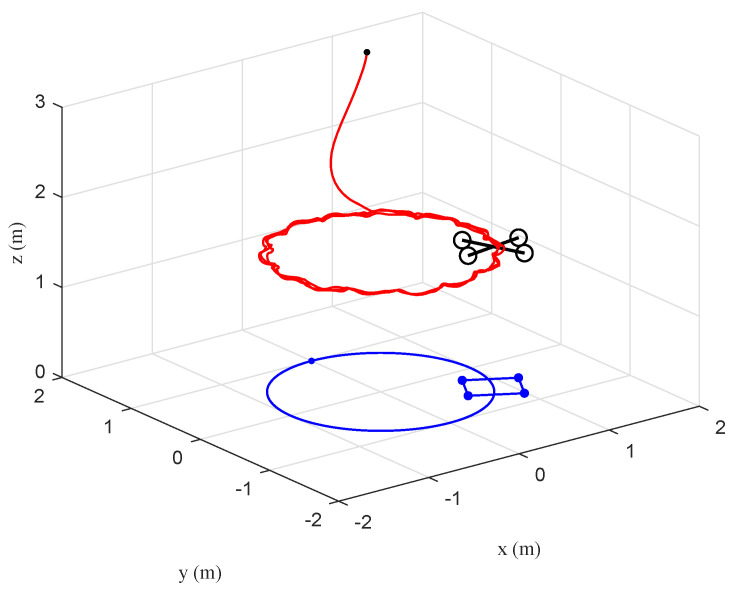
Scenario 2: Circular trajectory of the quadrotor and the target in a 3D environment under the proposed controller.

**Figure 13:**
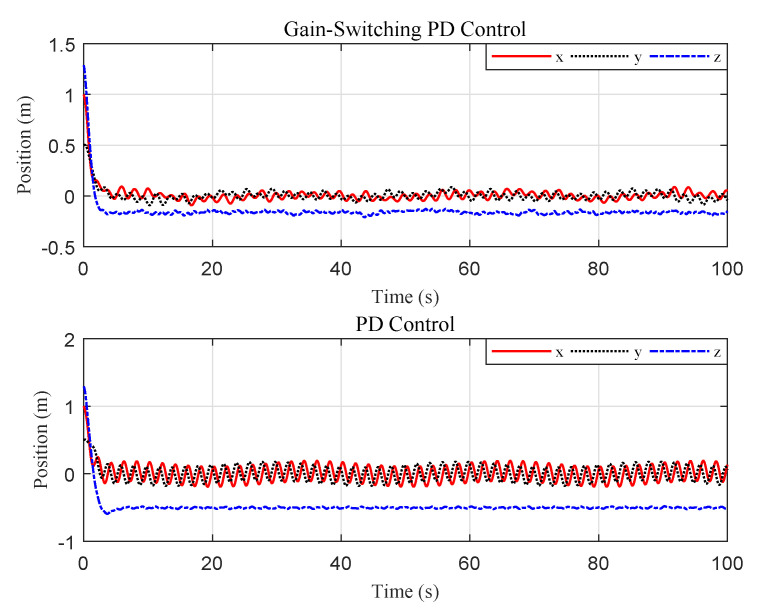
Scenario 2: Time evolution of the position tracking error under the two controllers (circular movement).

**Figure 14:**
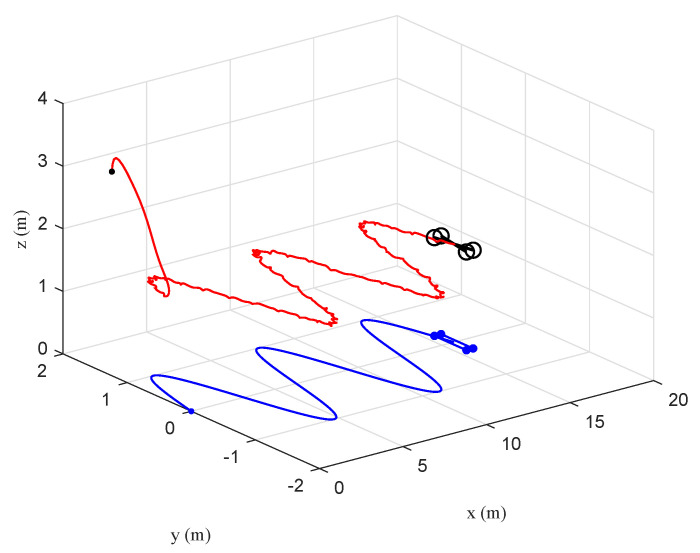
Scenario 2: S-type trajectory of the quadrotor and the target in a 3D environment under the proposed controller.

**Figure 15:**
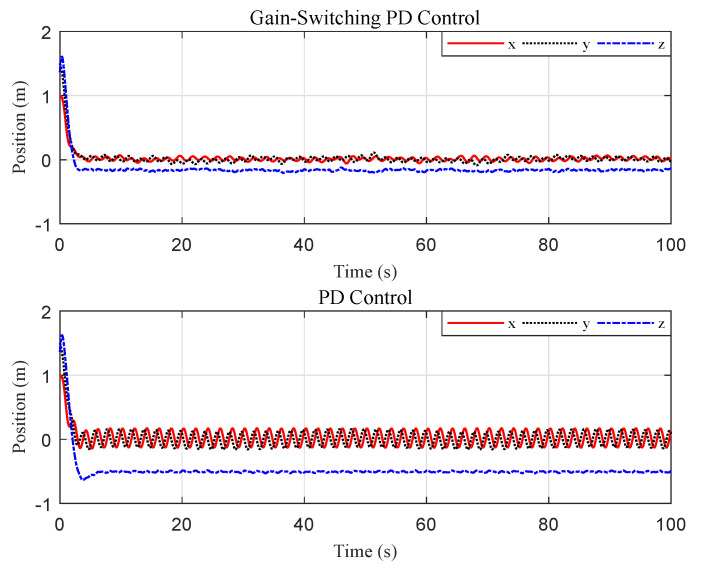
Scenario 2: Time evolution of the position tracking error under the two controllers (S-type movement).

**Figure 16:**
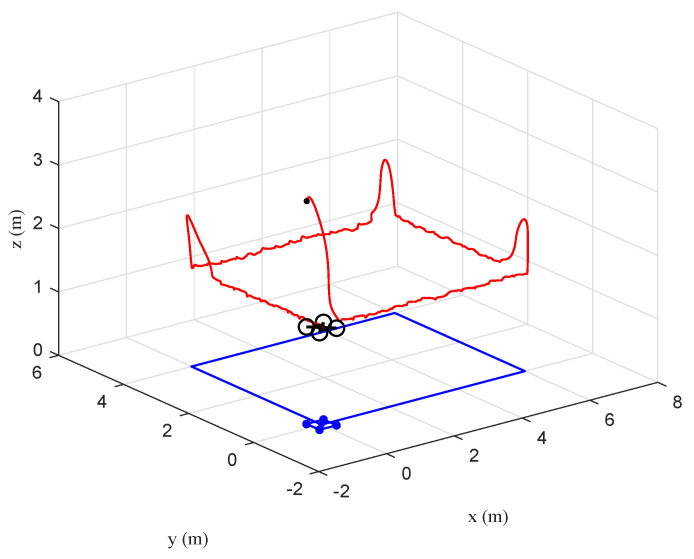
Scenario 2: Linear trajectory of the quadrotor and the target in a 3D environment under the proposed controller.

**Figure 17:**
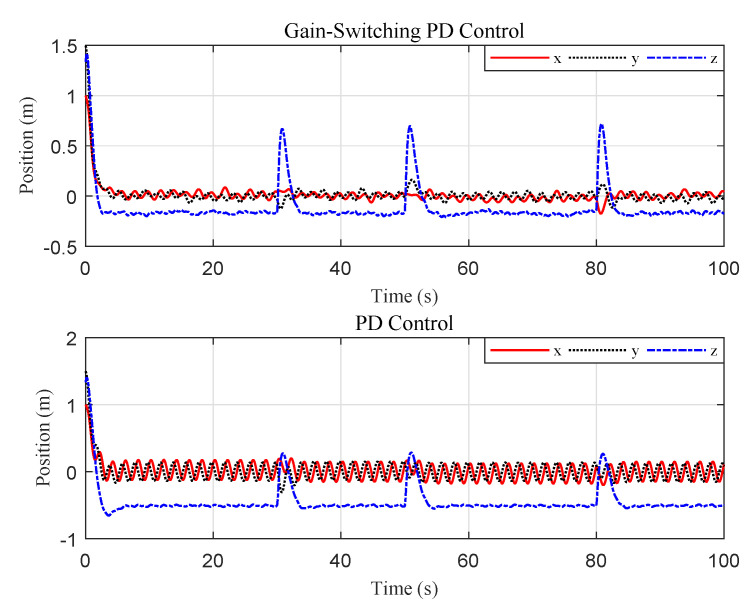
Scenario 2: Time evolution of the position tracking error under the two controllers (linear movement).

The tracking performance of the circular, S-type, and linear movements were illustrated in Figure 12, Figure 13, Figure 14, Figure 15, Figure 16 and Figure 17, respectively. Trajectories of motion of the quadrotor and the target in a 3D environment were plotted in Figure 12, Figure 14 and Figure 16, which showed satisfactory tracking performance in spite of the presence of disturbance and noise. Meanwhile, Figure 13, Figure 15 and Figure 17 depicted the position tracking error of the two controllers in different target movements. The results indicated that the proposed control strategy applied to different target maneuvers, and showed better robustness and higher tracking accuracy than the traditional PD controller. The only exception was that there existed some fluctuations during the linear tracking (Figure 16 and Figure 17). It was because of the vehicle’s reaction time when the target suddenly changed its direction.

## 6. Conclusions

In this paper, we have developed a vision-based control scheme of a quadrotor for target tracking in the absence of location information and geometric features of the target. After transforming the image features to a virtual camera frame, optical-based metrology is exploited to estimate the relative distance and velocity. At the same time, the height of the quadrotor and image size can be adjusted by regulating the optimal observation area and radius scaling factor. Considering the presence of external interference and model uncertainties, we presented a gain-switching proportional-derivative (PD) control strategy to improve the robustness of the system. Two case studies, corresponding to hovering and tracking scenarios, are presented in this work. The simulation results indicated that the proposed vision-based scheme performed better in both hovering and tracking missions, compared with the traditional PD control.

In future work, we are going to add a field of view constraint to the system, as the proposed algorithm can not guarantee that all visual features are always kept inside the field of view of the camera. We also plan to implement the proposed control scheme in a real quadrotor.

## Figures and Tables

**Table 1:** Control gains in the simulation.

Gains	Values	Gains	Values
$k_{P}$	1	$k_{P}^{\prime }$	8
$k_{D}$	1.44	$k_{D}^{\prime }$	4
$k_{\varepsilon }$	0.3	$k_{\varepsilon }^{\prime }$	1
$K_{P}$	diag{1.2,1.2,1.3}	$K_{P}^{\prime }$	diag{10,10,10}
$K_{D}$	diag{1.5,1.5,1.6}	$K_{D}^{\prime }$	diag{5,5,5}
$K_{\varepsilon }$	m· diag{0.3,0.3,0.5}	$K_{\varepsilon }^{\prime }$	$k_{\varepsilon }^{\prime }\cdot J$
ε	0.1	$\varepsilon^{\prime }$	0.3

**Table 2:** The steady-state performance (position error) of the two methods.

	Traditional PD Control			Gain switching PD Control		
	Mean	Amplitude	Accuracy	Mean	Amplitude	Accuracy
Initial Position (*m*) [1,0.8,−4]	8.9 ×$10^{-4}$	0.1475	0.3209	3.7 ×$10^{-4}$	0.0290	0.0628
	−0.0051	0.1360	0.2985	1.5 ×$10^{-4}$	0.0345	0.0731
	−0.5211	0.0164	0.5495	−0.1323	0.0082	0.1484
Initial Position (*m*) [1,2,−5]	9.0 ×$10^{-4}$	0.1475	0.3209	3.7 ×$10^{-4}$	0.0291	0.0628
	−0.0051	0.1360	0.2984	1.6 ×$10^{-4}$	0.0345	0.0731
	−0.5212	0.0165	0.5495	−0.1324	0.0083	0.1485
Initial Position (*m*) [0.6,0.8,−1.5]	8.8 ×$10^{-4}$	0.1475	0.3208	3.7 ×$10^{-4}$	0.0290	0.0627
	−0.0052	0.1360	0.2985	1.6 ×$10^{-4}$	0.0345	0.0731
	−0.5211	0.0164	0.5494	−0.1322	0.0082	0.1483
